# Clinical significances and features of prompt brain CT scan after intracranial artery stenting: analysis of 501 cases

**DOI:** 10.18632/oncotarget.23216

**Published:** 2017-12-14

**Authors:** Jie Li, Sai-Yu Cheng, Xiao-Yi Xiong, Chun-Mei Duan, Liang Liu, Yu Zhou, Jian-Rong Zhang, Li Wang, Kai Zhou, Zi-Li Gong, Yong Liu, Fei Wei, Jie Shuai, Lin Shen, Qing-Wu Yang

**Affiliations:** ^1^ Department of Neurology, Xinqiao Hospital & The Second Affiliated Hospital, The Army Medical University (Third Military Medical University), Chongqing, China

**Keywords:** prompt CT, intracranial artery stenting, ischemic stroke, double anti-platelet therapy

## Abstract

Cerebral hemorrhage is a serious complication of intracranial artery stenting that could be fatal without timely identification and treatment. Prompt brain CT scan would help to evaluate whether cerebral hemorrhage occurs, however, the diverse features of the CT scan immediately after stenting could influence the judgement sometimes. Therefore, we analyzed and summarized these features to help to determine the clinical significance of these CT features. The prompt CT features after stenting were classified into three types. Type I indicates that no high-density shadows. Type II indicates that high-density shadows scattered in the infarct areas and/or subarachnoid spaces without mass effect. Type III indicates high-density shadows scattered in and/or out of the infarct areas and/or subarachnoid space with obvious mass effects. Based on this classification, the patients in both Type I and II would continue the double anti-platelet treatment (DAPT) and anti-coagulation treatment, while the later need closer monitoring. However, patients in Type III must immediately withdraw the DAPT and anti-coagulation treatment with close monitoring and surgical intervention was needed when necessary. Nineteen (3.79%) patients were classified into Type III, and 5 (1.00%) of the 19 were accepted surgical intervention. Two of these patients died (0.40%). The prompt CT scan timely distinguishing the cerebral hemorrhage was necessary after intracranial artery stent angioplasty. Additionally, based on the different prompt CT features to take different therapeutic strategies after stenting would achieve better outcomes for ischemic stroke or transient ischemic stroke (TIA) patients underwent intracranial artery endovascular therapy.

## INTRODUCTION

Intracranial atherosclerosis is a major cause of ischemic stroke [1, 2]. Although studies showed that medication is an important secondary prevention and/or therapeutic strategy [2, 3], it alone cannot effectively reduce the development of stroke from severe intracranial atherosclerotic stenosis [4]. In light of this, revascularization of cerebral artery might be an important therapeutic method for the ischemic stroke and transient ischemic stroke (TIA) treatment and prevention with severe intracranial atherosclerotic stenosis. Recently, we and others have shown that intracranial artery stent angioplasty significantly improved the outcomes of patients with severe intracranial artery stenosis [5-8]. Therefore, endovascular therapy has been recognized as a novel and effective therapeutic method to prevent stroke and its recurrence [9]. However, the cerebral hemorrhage is the most serious complication during and after the procedure [10-14]. Moreover, as the patients need double anti-platelet treatments (DAPT) and anti-coagulation treatment during perioperative period [15-17], once the cerebral hemorrhage occurs and it can be fatal if not identified and treated promptly. Therefore, doctors would routinely administer computed tomography (CT) scans to observe brain conditions in the cerebral hemispheres about 30 minutes after endovascular therapy, which can promptly know whether the hemorrhagic occurrence. However, the prompt CT scan features following endovascular therapy are diverse that would cause difficulties to the timely diagnosis of bleeding. Therefore, quickly and accurately identify the clinical features of each CT scan would help neurologists to provide a best treatment plan for patients who underwent endovascular therapy.

In this study, we conducted a retrospective review of the experience in a single comprehensive stroke center to determine the clinical significance and feature of each CT scan in individuals who underwent endovascular treatment for ischemic stroke or TIA. Taking a summarization on the diverse CT features following endovascular therapy, and try to offer a best reference to clinical treatment.

## RESULTS

### Patient characteristics

The baseline clinical characteristics of the 501 patients recruited from June 2012 to April 2017 are shown in Table 1. Of the 501 subjects, 386 subjects (77.05 %) were received middle cerebral artery (MCA) stentings, 69 subjects (13.77 %) were received basilar artery (BA) stentings and 46 subjects (9.18 %) were received intracranial vertebral artery (V4) stentings. All the three groups had the similar high-risk factors of stroke, such as hypertension, hyperlipidemia, diabetes mellitus, and smoking. The mean diameter narrowing of intracranial artery was 82.83% ±6.76% in Type I, 81.34% ±5.36% in Type II, and 84.65% ± 3.8% in Type III. In addition, the mean stenting procedure time in MCA group were 93 min ± 14.3 min, 86 min ± 13.5 min in the BA group and 89 min ± 11.4 min in the V4 group. After stenting, the improved extent of stenosis was 75.7% ± 9.43% in MCA group, 73.6% ±7.56% in the BA group, and 74.2% ±8.25% in the V4 group, when compared to pre-operation.

**Table 1:** Baseline clinical characteristics and intervention results for the patients

Characteristics	Results		
	MCA (n = 376)	BA (n = 69)	V4 (n = 46)
Age (years), mean ± SD	62.4 ± 11.5	65.7 ± 13.1	63.5 ± 10.4
Male sex, n (%)	289 (76.9)	48 (69.6)	30 (65.2)
Hypertension, n (%)	247 (65.7)	43 (62.3)	31 (67.4)
Hyperlipidemia, n (%)	192 (51.1)	37 (53.6)	22 (47.8)
Diabetes mellitus, n (%)	148 (39.3)	23 (33.3)	17 (36.9)
Smoking, n (%)	198 (52.7)	38 (55.1)	24 (52.2)
History of previous stroke, n (%)	35 (9.3)	4 (5.8)	4 (8.7)
History of past use of antiplatelet strategies, n (%)	57 (15.2)	8 (11.6)	6 (13.0)
Stroke as a qualifying event, n (%)	205 (54.5)	37 (53.6)	25 (54.3)
Length of stenosis (>10 mm), n (%)	68 (18.1)	14 (20.3)	7 (15.2)
Seriously tortuous path, n (%)	54 (14.4)	7 (10.1)	6 (13.0)
Extent of stenosis before intervention (%), mean ± SD	84.3 ± 8.5	82.7 ± 7.6	83.1 ± 8.2
Extent of stenosis after balloon dilation (%), mean ± SD	34.2 ± 7.8	33.7 ± 8.2	35.3 ± 7.1
Extent of stenosis after intervention (%), mean ± SD	8.6 ± 3.5	9.1 ± 3.8	8.9 ± 2.9
Stenting procedure time (minutes), mean ± SD	93 ± 14.3	86 ± 13.5	89 ± 11.4

BA: basilar artery, F: female, M: male, MCA: middle cerebral artery, SD: standard deviation.

### Classification of prompt head CT scan features

**Figure 1:**
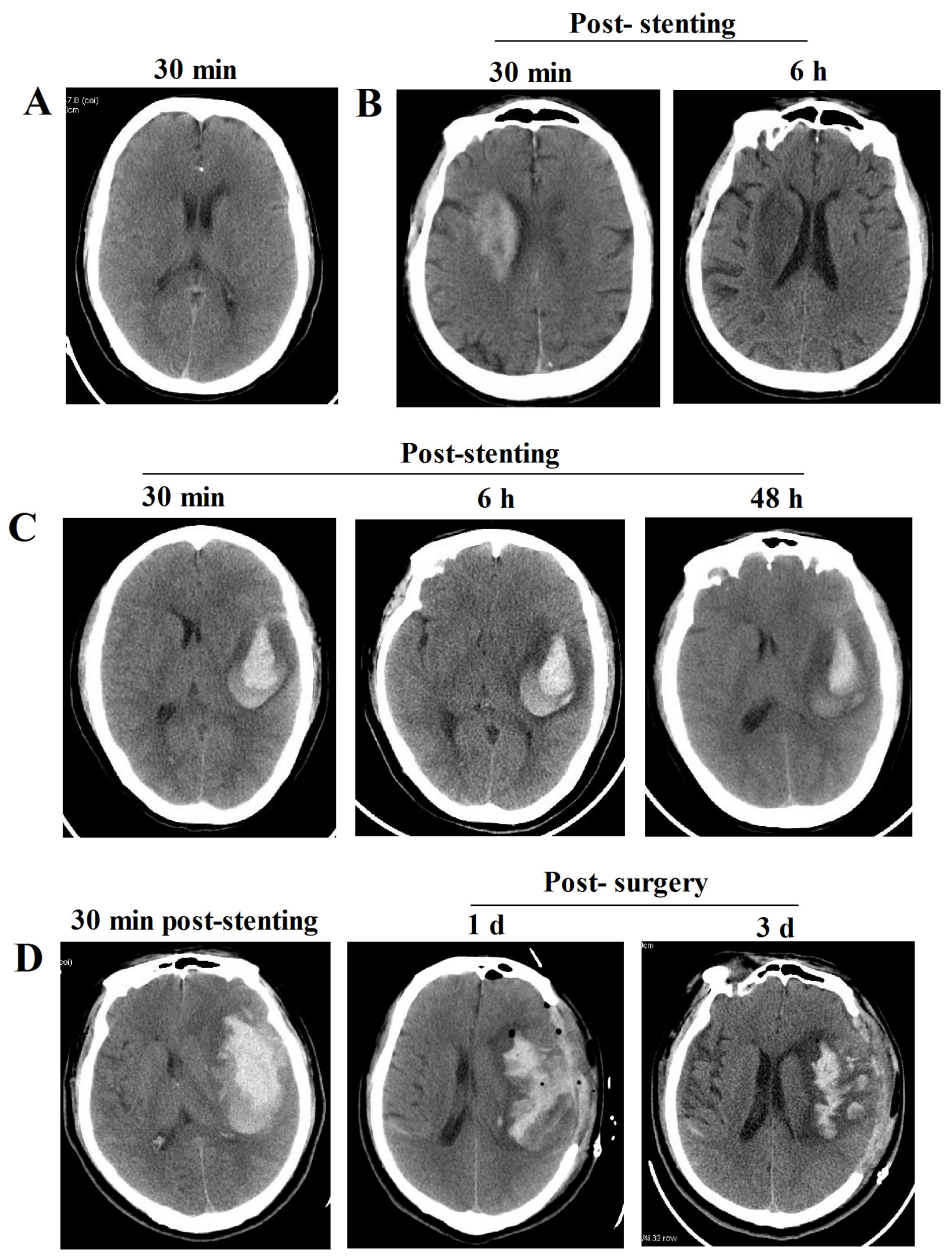
Representative images of the CT features in three Types after intracranial artery stenting **(A)** Representative CT image of patients in Type I. **(B)** Representative CT images of patients in Type II show that high-density shadows existed at 30 min after stenting were gradually disappeared within the next 24h. **(C)** Representative CT images of patients in Type IIIa show that high-density shadows occured at 30 min, which was not expanded at 6 h post-stenting, while the high-density shadow was shrank at 48 h after stenting. **(D)** Representative CT images of patients in Type IIIb show that high-density shadows existed at 30 min after stenting were gradually aggravated with obvious mass effects within next 6 h, then immediately receiving surgical operation.

The features of the prompt head CT scans exhibited high-density shadows distributed range from brain parenchyma, subarachnoid spaces and ventricle. No high-density shadows existed in prompt CT scans were classified into Type I (Figure 1A), and the CT value was 16.35 ± 8.76 Hu. Additionally, the clinical features of prompt CT scans after stenting in Type II and III were summarized in Table 2. In Type II, the CT value was 92.27 ± 114.96 Hu, and the scope of the high-density shadow was less than the infarction areas and without mass effects; most of the shadow was evenly distributed and scattered into brain parenchyma or subarachnoid spaces, and closing to cortex (Figure 1B). While in Type III, the CT value was 268.5 ± 323.0 Hu, and the scope of the high-density shadows exceeded the infarction areas and with obvious mass effects; the irregularity high-density shadows were bilateral sometimes and scattered into brain parenchyma, subarachnoid spaces and/or ventricle (Figure 1C, 1D).

**Table 2:** Clinical features of prompt CT scans in Type II and III

	High-density shadows existed in			
	BP	SP	BP and SP	BP, SP and ventricle
**Type II**	112 cases. Less than infarction areas without obvious mass effects	34 cases. Limited within the ipsilateral brain tissues and close to cortex	37 cases. Less than infarction areas without obvious mass effects	
**Type III**	1 cases. Exceeded the infarction areas with obvious mass effects		17 cases. Exceeded the infarction areas with obvious mass effects	1 cases. Exceeded the infarction areas with obvious mass effects. Most SAH were bilateral.

BP: brain parenchyma, SP: subarachnoid spaces, SAH: subarachnoid hemorrhage.

Based on this classification, we found that 221 cases in the MCA group were classified into Type I, 148 cases into Type II and 17 cases into Type III; while 60, 8, and 1 cases in the BA group were classified into Type I, II and III, respectively; and 18, 27 and 1 cases in the V4 group, respectively.

### The effectiveness of clinical strategies following endovascular therapy

**Figure 2:**
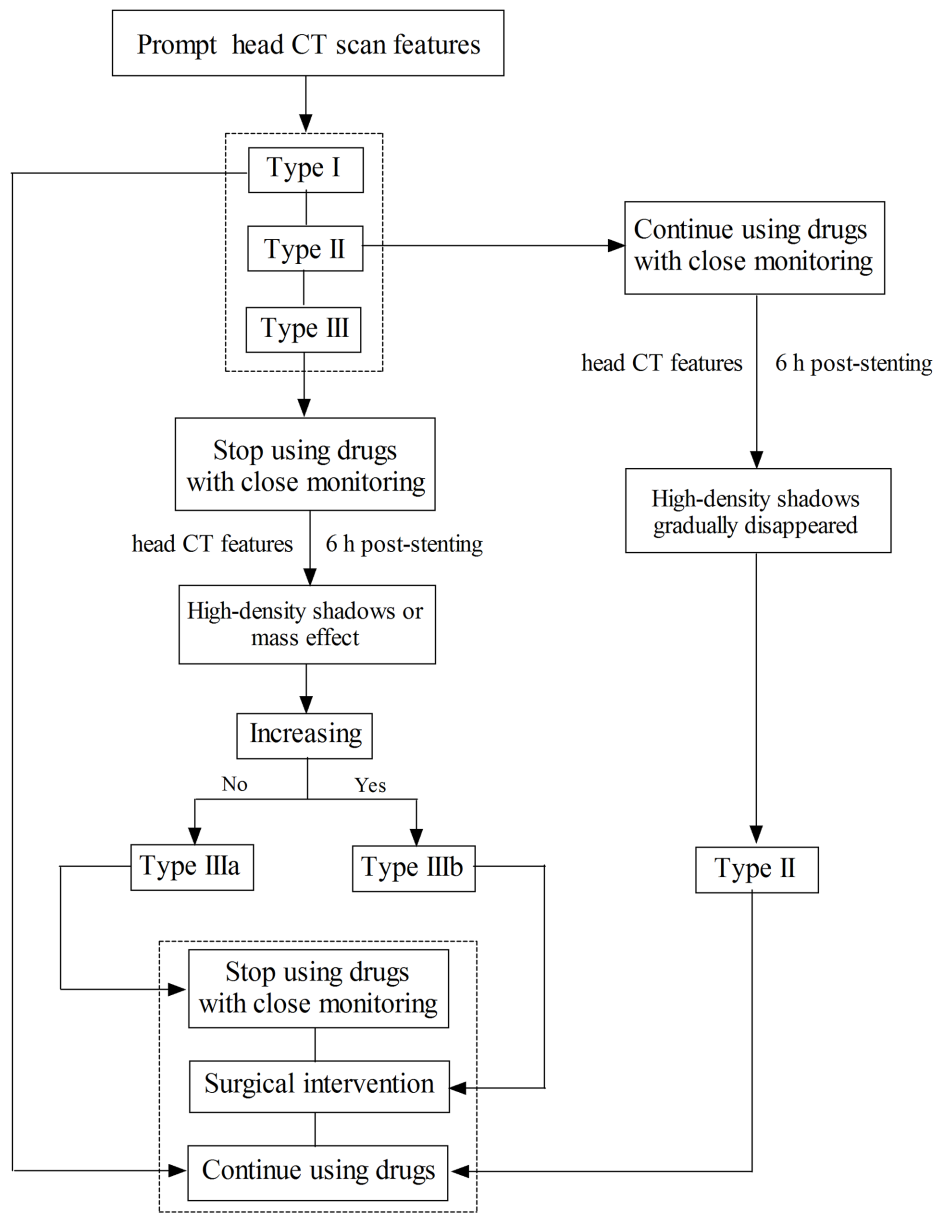
Flow chart

Figure 2 describes the evaluation procedure of the prompt CT features for the guidance of clinical treatment. If the prompt CT feature was Type I, the patients will continue regular anti-platelet (aspirin, 100 mg/day + clopidogrel, 75 mg/day, for 3 month) and anti-coagulation (enoxaparin, 40 mg/12 h, for 3 - 5 days) treatment; and when it was Type II, the patients will continue regular DAPT and anti-coagulation treatment with close monitoring based on the head CT reexamination; however, when it was Type III, the patients will first received single anti-platelet treatment (clopidogrel, 75 mg/d) with close monitoring when the 48-72 h post-stenting head CT scan showed that the hematoma was stabilized, if there was no expansion of hematoma within the next 5-7 d then added the other anti-platelet drug (aspirin, 100 mg/day) with close monitoring.

**Figure 3:**
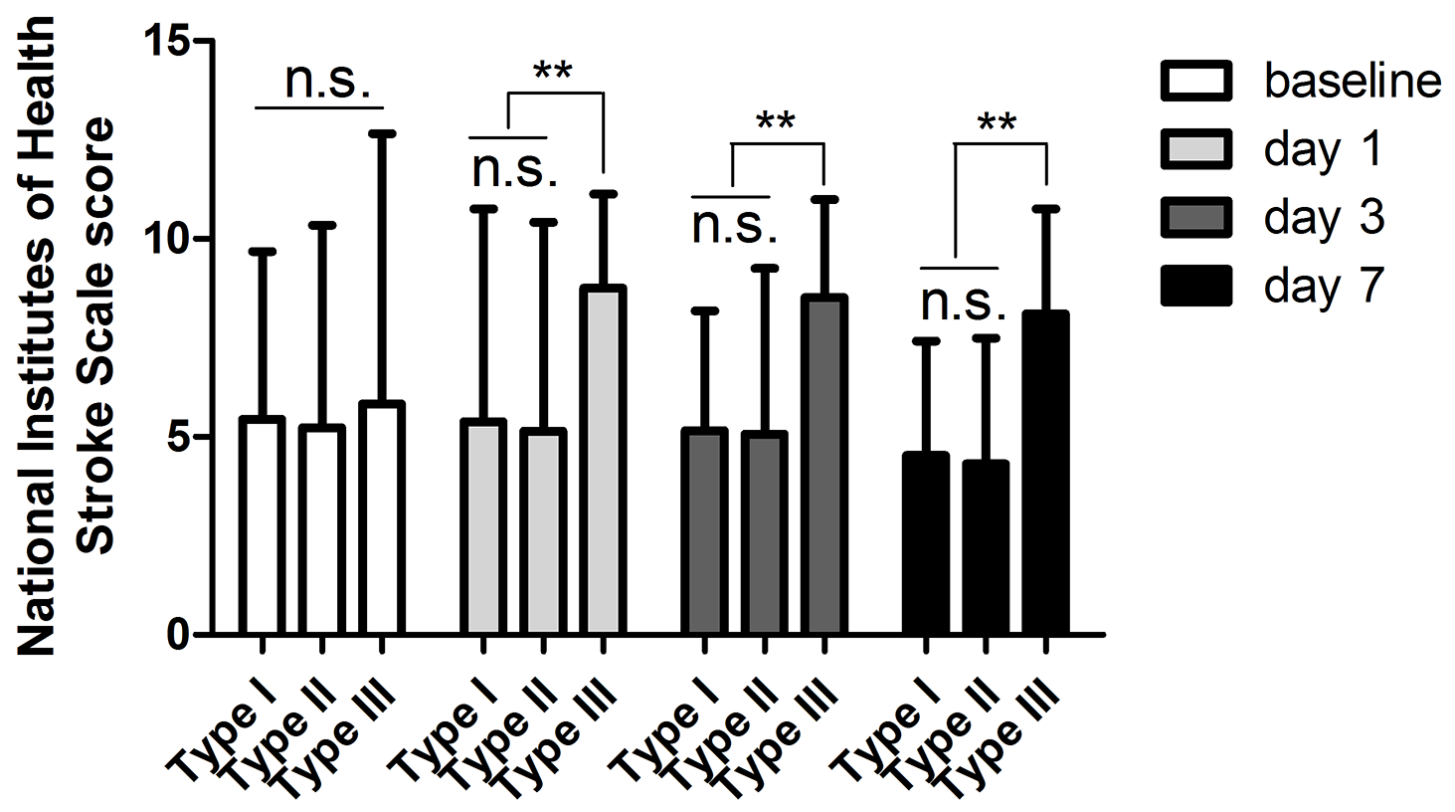
Better outcomes were obtained by comparing the NIHSS scores among the patients in Type I, Type II and Type III n.s indicates non-significance, ^**^p < 0.01.

Following this strategy, all of the patients in Type I and Type II were have a better outcome following the treatment strategy (Figure 3). In the Type IIIa, although the prompt head CT scan of the patients exhibited mass effects after stenting, 11 of 14 patients who received conservative treatment had a relative better outcome withdrawing the DAPT and anti-coagulation drugs the high-density shadows stopped enlarging, while 3 of them exhibited worsen symptoms when discharged (Supplementary Table 1). However, 2 of 5 cases in Type IIIb were dead after the stenting due to severe cerebral hemorrhage despite of receiving surgical and medical treatment, while the other 3 patients had a relative better outcome (Supplementary Table 1).

## DISCUSSION

In this study, we are the first to report that the clinical features and significances of the prompt head CT features after the intracranial artery stenting. We classified the prompt head CT features into three types that may help to determine whether continue to use the DAPT and anti-coagulation drugs after stenting. Except the patients classified into Type III have to withdraw the DAPT and anti-coagulation drugs immediately after stenting, the patients in other two types don’t have to, while in Type II the patients need close monitoring of the high-density shadows in the brain within 24 h. Following this strategy, we achieved better therapeutic effects although very few patients had worsened outcome or death that due to the severe cerebral hemorrhage.

We retrospectively found that 202 patients after stenting occurred high-density shadows exhibited by the prompt head CT scan. Among them, the scope of high-density shadows of 183 patients were less than infarction areas and gradually disappeared within the next 24 h (Type II), which might result from the leakage of contrast agent. While, in the other 19 patients, the scope was large than the infarction areas and would further aggravated and even with obvious mass effects within the next 24 h after stenting (Type III), which should be treated as cerebral hemorrhage with close monitoring.

The route of artery during the subarachnoid space is highly tortuosity and with many tiny perforated arteries before entering into the brain parenchymal, this anatomical feature is predisposed to let these arteries subject to different levels of endangium injury and perforation, rupture or tear during the endovascular operation, and may finally result in subarachnoid hemorrhage (SAH). In addition, postoperative hypertension was another important reason for cerebral hemorrhage after stenting. Once the intracranial arterial stenosis was eliminated, the blood would immediately flow into the distal of stenosis and may exceed the autoregulate ability of the brain and even lead to bleeding, because the impaired autoregulation as a result of endothelial dysfunction is implicated in the pathogenesis of CHS [20]. Therefore, control well the hypertension during the perioperation would be benefit for the prevention of CHS. In our study, we have strictly controlled the blood pressure under 120/80 mmHg to limit the increasing in cerebral perfusion, however there were still 19 patients occurred in cerebral hemorrhage. Additionally, recent cerebral infarction and high stenosis removal are considered to be the two most prominent factors to result in hyperperfusion syndrome [21, 22], because patients with severe bilateral carotid stenosis have been shown to be predisposed to ICH [23]. In our study, we also found that the stenosis of 19 patients happened to cerebral hemorrhage after stenting was 84.65% ± 3.8%. However, although the use of anti-platelet drugs was shown to not increase the risk ICH [23], the adequate usage during the perioperation may be a very negative factor for the treatment of patients happened to ICH. Therefore, in order to detect and treat the cerebral hemorrhage in time, prompt head CT was the first to be suggested to provide the information of brain parenchymal after stenting.

During the operation, 150 - 300 mL contrast agent was used to observe the information of arterial stenosis and stent. Therefore, the prompt head CT scan would exhibit diverse CT features, for instance, some normal brain structures (including the cerebral artery and the upper sagittal sinus) are clearly enhanced after stenting and will disappear within next 24 h, which was the normal brain CT features not the cerebral hemorrhage. And we classified this into Type I, patients in which will continue to DAPT and anti-coagulation after stenting. Additionally, no high-density shadows in brain parenchymal and subarachnoid spaces are also deemed to normal features. While those high-density shadows scattered in the infarct areas without mass effects were classified into Type II. Although these features should also not be considered as cerebral hemorrhage, at the meaning time the DAPT and anti-coagulation treatment were continued in our study, the patients were treated into intensive care unit for 24 h with close monitoring. The head CT scan should be arranged at the next 6 h after stenting to further observe the situation of high-density shadows. Once the high-density shadows were increasing and/or occurred in mass effects and/or additional neurological signs, the DAPT and anti-coagulation drugs must be withdrawal, further head CT scan should be rearranged at the following 24 h after stenting based on the patients’ situation. If the prompt head CT scan exhibited high-density shadows with obvious mass effects, then immediately classified into Type III and withdraw all the DAPT and anti-coagulation drugs. Regarded to this type, previous reports showed that patients will receive the DAPT only after the hematoma was completely absorbed [24, 25]. While we first continued single anti-platelet (clopidogrel, 75 mg/d) treatment with close monitoring when the hematoma was stabilized showed by the 48-72 h post-stenting head CT scan, then we added the other anti-platelet drug (aspirin, 100 mg/day) with close monitoring if there was no expansion of hematoma within the next 5-7d (Figure 1C), because the DAPT was also critical important for the treatment of patients who underwent stenting ^8^. The data analysis found that our therapeutic strategy did not increase the re-bleeding risk, because 11 of 14 patients who received conservative treatment in Type IIIa had a relative better outcome although they experienced cerebral hemorrhage after stenting. However, when the cerebral hemorrhage companied with the tendency of brain hernia formation, the surgical intervention was suggested to save their life. It is a pity that 2 patients were dead although they received the surgical intervention due to the uncontrolled hemorrhage.

In summary, cerebral hemorrhage is a potentially devastating complication of intracranial arteries revascularization after stenting. Therefore, the prompt head CT scan was the first choice to observe the intracranial condition, however, the CT features are rather diverse and would easily mislead the neurologists. Then, our classification of these diverse prompt head CT features into three types might help clinical neurologists provide better guidance for patients after stenting.

## MATERIALS AND METHODS

### Patients

We retrospectively reviewed a prospective register of 501 patients who underwent endovascular therapy for ischemic stroke or TIA at the Xinqiao Hospital, the Army Medical University (Third Military Medical University) in Chongqing, China, from June 2012 to April 2017. The protocol of this retrospective analysis for endovascular treatment were approved by the ethics committee of Xinqiao Hospital.

All eligible patients met the following criteria: aged 18 years or older and had a symptomatic intracranial atherosclerotic stenosis (ICAS) of 70% to 99% with a lesion length of ≤15 mm and target vessel diameter of ≥2.0 mm in the anterior or posterior intracranial arteries (including middle cerebral artery, intracranial vertebral artery and basilar artery). The measurements were made on digital subtraction angiography (DSA) using a previously reported method [18]. The symptoms could be TIA or stroke within the past 90 days and ascribed to hypoperfusion in the culprit vessel territory. According to Miao et al. [19] reported, hemodynamic impairment in the territory of the culprit artery was determined on imaging within 2 weeks before the operation. Informed consent was signed by patients or their direct family members. The images were centrally reviewed by 3 experienced physicians, and the disagreement were allowed to be resolved by discussion.

Patients were excluded if they were current pregnancy or lactation, or had cerebral hemorrhage, cerebral arteriovenous malformations, aneurysms, and bilateral moderate-severe carotid stenosis, or had acute infarcts within 3 weeks, severe vessel tortuosity precluding the deployment of endovascular devices as determined by the executive committee, nonatherosclerotic lesion confirmed by high-resolution magnetic resonance imaging (MRI), embolic or perforator stroke based on MRI or CT, or baseline disability. Only patients without risk factors for intracranial atherosclerosis, or patients with lesion suspected to be nonatherosclerotic by regular CT, MRI, or DSA, were subjected to high-resolution MRI.

### Preoperative evaluation and medical treatment

According to the guidelines of ischemic stroke diagnosis and treatment (China), the ischemic stroke was confirmed by the diffusion weighted sequence of MRI. TIA was diagnosed with the criteria that patients display a sudden-onset focal cerebral or retinal neurological deficit of possible vascular etiology and lasting less than 24 hours and without abnormal brain image confirmed by MRI. The diagnostic digital subtraction angiography (DSA) or CT angiography (CTA) and CT perfusion (CTP) were arranged during the hospitalization or Stroke center to evaluate the ischemic degree of the anterior or posterior cerebral artery. Before the endovascular therapy, all the patients would receive CTP examination to assess cerebral circulation reserve. All the patients received normal medical treatment, and patients started receiving aspirin (100 mg/d) and clopidogrel (75 mg/d) daily three to five days before stenting or a loading dose of 300 mg clopidogrel if the procedure was considered urgent. They were maintained on aspirin (100 mg/d) and clopidogrel (75 mg/d) for 90 days after stenting. The anti-coagulation treatment (enoxaparin, 40 mg/12h) would last 3 to 5 days after stenting. Hyperlipidemia was treated to maintain low-density lipoprotein (LDL) levels at less than 70 mg/dL (1.81 mmol/L) or a decrease by 50%. Those patients with freshly ischemic lesion and massive cerebral infarction needed medical treatment for at least 3 weeks to 1 month before stenting.

### Endovascular stenting procedure

Three experienced surgeons who had more than 100 cases of intracranial stent implantation performed the stenting surgeries. All procedures were performed under general anesthesia to avoid the patients’ head movement. Intravenous heparin was administered after the placement of vascular access using a bolus of 75 U/kg followed by half of the dose 1 hour later. Femoral artery puncture was performed using the Seldinger technique to place a 6F/8F arterial sheath. A 6F guiding catheter (Cordis Corporation, Miami, FL) was delivered to the C2 segment of the internal carotid artery or V1 segment of vertebral artery. Microcatheter insertion combined with guidewire exchange was performed to place the guidewire in the distal end of the stenotic vessel. A gateway balloon (Stryker, Maple Grove, MN) was placed across the stenotic segment for balloon dilation. Then, the balloon was removed, and the Wingspan stent (Stryker, Maple Grove, MN) was placed in the lesioned vessel. After radiographic examination revealed that the degree of stenosis had significantly improved, the stent delivery system was removed. 150 - 300 mL Iodixanol (320 mg I/mL) was used during operation as contrast agent. Following 15 - 30 minutes’ postoperative observation, intracranial angiography was repeated, the surgery was completed if no abnormalities were observed, then the patients received the head CT scan once at 30 min and/or several times when necessary within the next 24 h after stenting. The patients were then admitted to intensive care unit for 24 h with close monitoring. Antihypertensive agents were used to maintain systolic pressure lower than 120 mmHg and diastolic pressure lower than 80 mm Hg. The National Institutes of Health Stroke Scale (NIHSS) score of these patients was obtained at baseline, day 1, day 3 and day 7 after stenting.

### Head CT scan

Non-contrast head CT scan was performed once immediately after stenting, or several times within 24 h and even 7 days after stenting based on the clinical symptoms of the patients. The brain images of CT examinations were performed using a second-generation dual-source CT scanner (Lightspeed-16, General Electric Company, USA). The CT parameters are defined as follows: Iamges: 1-28, CTDlvol: 40.46mGy, DLP: 566.46mGy/cm, Dose Eff: 97.40%, Scan Type: Axial, Desired KV: 120, Manual mA: 220, Thickness mm: 5.0, Interval mm: 20, DFOV cm: 25.0, Window Width: 90 Hu, Window Level: 40 Hu.

### The classification of prompt head CT scan features

According to the diverse features of prompt head CT scan after stenting, we classified these into three types (Table 3). Type I indicates that prompt head CT scans without high-density shadows. Otherwise, they would be classified into Type II and III when high-density shadows existed. If the shadows were gradually disappeared in the reexamination CT scans within 24 h after stenting, which would be classified into Type II; while Type III indicates that the shadows were unabsorbed or enlarged in the reexamination CT scans within 24 h after stenting.

**Table 3:** Classifications of the prompt head CT features

**Type I (Normal)**
The prompt CT features were consistent with pre-operation without high-density shadows or some normal brain structures (including the cerebral artery and the upper sagittal sinus) are clearly enhanced
**Type II (Contrast agent exudation)**
High-density shadows scattered in the infarct areas and/or subarachnoid spaces, which was gradually dismissed in the head CT features within 24 h post-stenting; without mass effects.
**Type III (Cerebral hemorrhage)**
High-density shadows scattered in and/or out of the infarct areas, with high-density shadows in the subarachnoid space; obvious mass effects.
** IIIa (Stop the DAPT and anti-coagulation treatment with close monitoring)**
Multiple post-operation head CT features showed that high-density shadows were not increasing during 24 h after stenting.
** IIIb (Surgical intervention)**
Multiple post-operation head CT features showed that high-density shadows were still increasing, and the mass effect was increasing during 24 h after stenting.

DAPT: double anti-platelet therapy, CT: computed tomography.

### Statistical analysis

Continuous variables are presented as mean ± standard deviation (SD). Categorical variables are presented as numbers and percentages. The NIHSS scores among the Type I, Type II and Type III during the peri-operation time were compared using the two-way ANOVA test. *P* < 0.05 was considered statistically significant. All statistical analyses were performed using SPSS for Windows (Version 16.0, SPSS Inc., Chicago, IL, USA).

## SUPPLEMENTARY MATERIALS TABLE


