# Supplementary material for: Clinical significances and features of prompt brain CT scan after intracranial artery stenting: analysis of 501 cases
Source: Oncotarget. 2017 Dec 14;8(69):114259–67. doi: 10.18632/oncotarget.23216 (PMC5768401; doi:10.18632/oncotarget.23216)
Supplement: Supplementary file 1 [file oncotarget-08-114259-s001.pdf]

Clinical significances and features of prompt brain CT scan after intracranial artery stenting: analysis of 501 cases

SUPPLEMENTARY MATERIALS

Supplementary Table 1: Clinical features of patients in Type III.

No.	Sex	Age (year)	Stenting position	Length of stenosis(mm)	Extent of stenosis before intervention(%)	Extent of stenosis after intervention(%)	Type	NHISS at admission	NHISS at discharge
1#	M	71	LM	12.13	96.87	12	IIIa	0	5
2#	F	49	LM	4.1	85.93	7.1	IIIa	0	6
3#	M	59	RM	11.25	83.95	8.5	IIIa	10	10
4#	F	78	RM	9.52	84.76	10.4	IIIa	6	6
5#	M	62	BA	2.16	76.73	12.6	IIIa	8	8
6#	M	56	LM	4.25	83.52	8.8	IIIa	12	12
7#	M	50	LM	10.01	90.38	7.5	IIIa	15	15
8#	M	71	RM	7.02	78.89	8.5	IIIa	8	8
9#	M	62	LM	6.52	89.67	10.2	IIIa	6	6
10#	F	62	RM	3.45	77.01	9.5	IIIa	10	8
11#	M	68	RM	8.21	85.24	7.6	IIIa	4	7
12#	F	65	LM	4.75	80.75	7.2	IIIa	8	8
13#	F	71	LM	7.28	79.01	8.8	IIIa	5	5
14#	M	56	RM	8.12	86.53	7.1	IIIa	7	7
15#	M	66	LM	5.86	83.87	11	IIIb	9	7
16#	M	58	LM	12.05	82.35	9.2	IIIb	0	11
17#	M	49	RM	2.86	90.07	6.3	IIIb	4	9
18#	M	67	RM	9.35	73.9	7.1	IIIb	0	Death
19#	M	58	RV4	9.2	99	10.6	IIIb	0	Death

M: male, F: female, NIHSS: National Institutes of Health stroke scale, LM: left middle cerebral artery, RM: right middle cerebral artery, BA: basilar artery, RV4: right V4 segment of vertebral artery
